# A Doctor’s Don’ts

**Published:** 1890-03

**Authors:** 


### A DOCTOR'S DON'TS.

---

Don't read in street cars or other jolting vehicles.

Don't pick the teeth with pins or any other hard substance.

Don't neglect any opportunity to insure a variety of food.

Don't eat or drink hot and cold things immediately in succession.

Don't pamper the appetite with such variety of food that may lead to excess.

Don't read, write or do any delicate work unless receiving the light from the left side.

Don't direct special, mental or physical energies to more than eigpt hours' work in each day.

Don't keep the parlor dark unless you value your carpet more than you and your children's health.

Don't delude yourself into the belief that you are an exception as far as sleep is concerned; the nominal average of sleep is eight hours.

Don't endeavor to rest the mind by absolute inactivity; let it seek its rest in work in other channels, and thus rest the tired part of the brain.—*Richmond, Va., State.*

